# Serum ferritin associated with atherogenic lipid profiles in a high-altitude living general population

**DOI:** 10.7717/peerj.19104

**Published:** 2025-03-24

**Authors:** Menglong Jin, Mawusumu Mamute, Hebali Shapaermaimaiti, Hongyu Ji, Zichen Cao, Sifu Luo, Mayire Abudula, Abuduhalike Aigaixi, Zhenyan Fu

**Affiliations:** 1 Department of Cardiology, State Key Laboratory of Pathogenesis, Prevention and Treatment of High Incidence Diseases in Central Asia, the First Affiliated Hospital of Xinjiang Medical University, Urumqi, Xinjiang, China; 2 Department of Urology, First People’s Hospital of Kashgar District, Kashgar, Xinjiang, China; 3 Disease Control and Prevention Center of Tashkurgan Tajik Autonomous County, Tashkurgan, Xinjiang, China; 4 Health Commission of Tashkurgan Tajik Autonomous County, Tashkurgan, Xinjiang, China

**Keywords:** High altitude, Atherogenic lipid profiles, SdLDL-C, Lp(a), Serum ferritin

## Abstract

**Background:**

Serum ferritin (SF) levels are associated with metabolic syndrome and dyslipidemia. However, the association between SF and atherogenic lipid profiles in high-altitude living populations remains unclear.

**Methods:**

In 2021, a cross-sectional study was conducted on adult Tajik individuals residing in Tashkurgan Tajik Autonomous County (average altitude 3,100 meters). Demographic information and anthropometric measurements were collected in local clinics. Fasting blood samples were analyzed using a Beckman AU-680 Automatic Biochemical analyzer at the biochemical laboratory of Fuwai Hospital. Univariate linear regression analyses were used to explore the association between SF and atherogenic lipid levels. Subgroup analysis was used based on gender and different high-sensitivity C-reactive protein (hs-CRP) and serum amyloid A (SAA) levels. The association between higher SF quartiles and different kinds of dyslipidemia were analyzed by logistic regression.

**Results:**

There were 1,703 participants in total, among which 866 (50.9%) being men. The mean ages of male and female participants were similar (41.50 *vs.* 42.38 years; *P* = 0.224). SF levels were significantly correlated with total cholesterol (TC) (Beta = 0.225, *P* < 0.001), low-density lipoprotein cholesterol (LDL-C) (Beta = 0.197, *P* < 0.001), high-density lipoprotein cholesterol (HDL-C) (Beta = −0.218, *P* < 0.001), triglycerides (TG) (Beta = 0.332, *P* < 0.001), and small dense LDL-C (sdLDL-C) (Beta = 0.316, *P* < 0.001), with the exception of lipoprotein (a) (Lp(a)) (Beta = 0.018, *P* = 0.475). SF was significantly correlated with LDL-C and HDL-C in women, and correlated with TC, TG, and sdLDL-C levels in both men and women in different inflammatory conditions. Elevated SF levels was significantly correlated with high TC (OR: 1.413, 95% CI [1.010–1.978]), high TG (OR: 1.602, 95% CI [1.299–1.976]), and high sdLDL-C (OR: 1.631, 95% CI [1.370–1.942]) in men and high TC (OR: 1.461, 95% CI [1.061–2.014]), high LDL-C (OR: 2.104, 95% CI [1.481–2.990]), low HDL-C (OR: 1.447, 95% CI [1.195–1.752]), high TG (OR: 2.106, 95% CI [1.454–3.050]), and high sdLDL-C (OR: 2.000, 95% CI [1.589–2.516]) in women. After adjusting for potential confounders, elevated SF levels continue to be correlated with high TG in male (OR: 1.382, 95% CI [1.100–1.737]) and female (OR: 1.677, 95% CI [1.070–2.628]) participants. In both young and middle-aged subgroups, the associations between SF and TG, TC, HDL-C, LDL-C, and sdLDL-C were still significant.

**Conclusions:**

SF was closely related to atherogenic lipid profiles, especially with regard to TG in high-altitude populations. This association cannot be attributed to its role as an inflammation marker.

## Introduction

Globally, cardiovascular disease (CVD) ranks as the top cause of death (Arnett et al., 2019; Visseren et al., 2021). Low-density lipoprotein cholesterol (LDL-C) is a recognized independent factor contributing to CVD risk and a primary target for CVD prevention (Mach et al., 2020). A subset of LDL, known as small dense LDL (sdLDL), holds a particularly strong association with the risk of atherosclerotic CVD (ASCVD) (Krauss, 2022; Yanai et al., 2022). Notably, lipoprotein a (Lp(a)) has been proven as a causal risk factor for ASCVD, independent of LDL-C (Reyes-Soffer et al., 2022). Additionally, it has been found that triglycerides (TG) and triglyceride-rich lipoproteins (TGRL) play an important in the residual risk realated to ASCVD (Dhindsa et al., 2020).

Inflammation has various maladaptive functions that lead to the progression and instability of ASCVD, and high-sensitivity C-reactive protein (hs-CRP) serve as a main clinical indicator of inflammation (Lawler et al., 2021). Furthermore, serum amyloid A (SAA) and serum ferritin (SF) are also important in acute and chronic inflammation, and are used as additional markers of inflammatory conditions (Kell & Pretorius, 2014; Zhang et al., 2019).

SF, apart from being an inflammation marker, is also vital in the iron delivery system and is gauged as an indicator of iron status (Kell & Pretorius, 2014; Lopez et al., 2016). People who live at high altitudes for a long time are constantly in a hypoxic environment. Erythrocytosis is an important adaptation mechanism that requires substantially higher amounts of iron (Gassmann & Muckenthaler, 2015). A study on young Swiss male whose residential altitudes range from 200 to 2,000 m revealed that for every 300 m increase in residential altitude, the levels of hemoglobin (Hb) and SF increased significantly (Staub et al., 2020). However, a study conducted in Han Chinese males showed that SF levels of participants living at high altitudes did not increase compared with those of sea level residents (Liu et al., 2018). The iron stores of high-altitude residents were distinct among different populations. In healthy people, iron stores were in the physiological range in spite of the increased need for erythropoiesis. However, for vulnerable groups living at a high altitude with a greater demand for iron, their iron stores were not likely to be quickly replenished as in normal situations (Böning et al., 2004; Burke et al., 2017; Cook et al., 2005; Tufts et al., 1985).

Preliminary studies conducted in different populations, including Spanish (González et al., 2006), American (Li et al., 2023), Qatar (Al Akl et al., 2021), and Chinese (Li et al., 2017; Zhou, Liu & Yuan, 2021) adults as well as Korean (Kim et al., 2016) and Chinese (Zhu et al., 2019) adolescents, have demonstrated that SF was significantly associated with dyslipidemia. Nevertheless, the association between SF and atherogenic lipid profiles in high-altitude living populations was unclear. Given the remarkable effect of high altitude on iron stores and SF levels, studies investigating the correlations between SF and dyslipidemia specifically in high-altitude residents are necessary. To fill this knowledge gap, we investigate for the first time the association between SF and atherogenic lipid profiles in a general population of Tajik adults living on the Pamirs Plateau of China.

## Materials & Methods

### Study population

In 2021, we conducted a cross-sectional study in northwestern Xinjiang, China. The eligible participants were Tajik individuals aged 18 years or older residing in Tashkurgan Tajik Autonomous County, which boasts an average altitude of 3,100 m. A total of 1,830 individuals were initially recruited for this study, with 1,753 completing the survey. Subsequently, 50 participants with a history of stroke, chronic inflammatory diseases, coronary heart disease (CHD), or those currently on lipid-lowering drugs were excluded. Ultimately, 1,703 participants were incorporated into the analysis. All participants provided written informed consent, and the study was approved by the Ethics Committee of the First Affiliated Hospital of Xinjiang Medical University (210521 - 02).

### Data collection

Sociodemographic information (age, gender, educational background, smoking habits, alcohol consumption, and disease history) was collected through questionnaires. After a 5-minute rest, two blood pressure (BP) measurements were made. The means of these two readings was employed for analysis. We measured waist circumference (WC), height, and body weight when participants were in lightweight clothing and without shoes. We calculated body mass index (BMI) as follows: BMI (kg/m^2^) = weight (kg)/(height (m))^2^. Blood samples were collected after an 8–14 h overnight fast as recommended by the World Health Organization (Alberti & Zimmet, 1998). These blood samples were then centrifuged at 3,000 rpm for 5 min, after which the serums were rapidly frozen at −80 degrees Celsius. Subsequently, the samples were transported to the biochemical laboratory of the Department of Epidemiology of Fuwai Hospital. This laboratory was part of the Lipid Standardization Program run by the US Centers for Disease Control and Prevention, ensuring the accuracy of tests conducted. Fast blood glucose (FBG), TC, TG, high-density lipoprotein cholesterol (HDL-C), Lp(a), sdLDL-C, ferritin, hs-CRP, and SAA were tested using a Beckman AU-680 Automatic Biochemical analyzer. The Friedewald formula was used to calculate LDL-C. LDL-C (mmol/L) = TC (mmol/L)−HDL-C (mmol/L)−TG (mmol/L)/2.2. The collection of questionnaires, physical examinations, and blood samples was carried out by trained staff at the local health center.

### Outcome definition

We used the 2023 Chinese guidelines for the management of dyslipidemia in adults to define different types of dyslipidemia (Joint Committee on the Chinese Guidelines for Lipid Management, 2023). High TC, high LDL-C, high TG, low HDL-C, high Lp(a), and high sdLDL-C were defined as TC ≥ 6.22 mmol/L, LDL-C ≥ 4.14 mmol/L, TG ≥ 2.26 mmol/L, HDL-C < 1.04 mmol/L, Lp(a) > 300 mg/L, and sdLDL-C ≥ 1.4 mmol/L, respectively. Smoking was defined as smoking at least 100 cigarettes and smoking within the last month. Drinking was defined as drinking at least 12 times in the last year. Exercise was defined as engaging in moderate or vigorous physical activity ≥ 150 min per week.

### Statistical analyses

IBM SPSS Statistics 26 was used to do statistical analyses. Continuous variables were shown as means ± standard deviation (SD) (normal distribution) or as quartiles (non-normal distribution). Category variables were represented as numbers (percentages). T-tests were utilized to evaluate the differences in continuous variables that followed a normal distribution, while Mann–Whitney U tests were employed for those that did not adhere to a normal distribution. Chi-square tests were utilized to investigate differences among category variables. Standardized regression coefficients (Beta) calculated by univariate linear regression analyses were used to assess the correlation between SF levels and lipids. Univariate logistic regression analyses were used to test the trend of dyslipidemia along ferritin. Multivariate logistics regression analyses were used to exclude the influence of confounder on dyslipidemia. *P* < 0.05 was considered statistically significant.

## Results

### Population characteristics

Among the 1,703 participants, 866 (50.9%) were men. The average ages of men and women were similar (41.50 *vs* 42.38 years; *P* = 0.224). The mean hemoglobin levels of Tajik reached up to 158.88g/L with a significantly gender discrepancy (168.17 *vs* 149.28 g/l; *P* < 0.001). Male participants had larger WC (mean 80.83 *vs* 76.16 cm; *P* < 0.001), slower heart rates (mean 65.11 *vs* 67.94 times/minute; *P* = 0.001), and higher rates of smoking (36.3% *vs* 0.1%; *P* < 0.001) and drinking (14.5% *vs* 0.1%; *P* < 0.001) than females, but the age, BMI, BP, and prevalence of hypertension and diabetes were similar. Regarding inflammation markers, men had lower SAA levels (median 5.30 *vs* 5.50 mg/L; *P* < 0.001) and notably higher SF levels (median 200.20 *vs* 42.30 ng/mL; *P* < 0.001) than women. There was no significant difference in hs-CRP concentration between men and women (median 4.28 *vs* 4.58 mg/L; *P* = 0.098). In terms of lipid profiles, men had higher TG (median 1.05 *vs* 0.84 mmol/L; *P* < 0.001), ApoB (mean 0.75 *vs* 0.72 g/L; *P* = 0.001), and sdLDL-C (mean 37.63 *vs* 32.85 mmol/L; *P* < 0.001) concentration but lower HDL-C (mean 1.18 *vs* 1.37; *P* < 0.001) and ApoA1 (mean 1.19 *vs* 1.29 g/L; *P* < 0.001) concentration than women (Table 1).

**Table 1:** Demographics and baseline characteristics of participants.

	Total (*n* = 1703)	Men (*n* = 866)	Women (*n* = 837)	*P* value
Age (years)	41.93 ± 14.83	41.50 ± 14.78	42.38 ± 14.89	0.224
Hypertension N (%)	359(21.1)	170(19.6)	189(22.6)	0.136
Diabetes N (%)	33(1.9)	22(2.6)	11(1.3)	0.066
Smoking N (%)	307(18.0)	306(36.3)	1(0.1)	<0.001
Drinking N (%)	123(7.2)	122(14.5)	1(0.1)	<0.001
Exercise N (%)	216(12.7)	160(19.0)	56(6.7)	0.079
WC (cm)	78.54 ± 14.74	80.83 ± 15.48	76.16 ± 13.54	<0.001
BMI (kg/cm2)	22.25 ± 12.69	21.91 ± 3.16	22.60 ± 17.81	0.266
SBP (mmHg)	120.18 ± 24.84	119.82 ± 23.12	120.56 ± 26.52	0.542
DBP (mmHg)	74.70 ± 15.90	74.55 ± 15.16	74.85 ± 16.64	0.701
HR (times/minute)	66.50 ± 16.86	65.11 ± 15.83	67.94 ± 17.77	0.001
FBG (mmol/L)	4.89 ± 1.19	4.91 ± 1.01	4.86 ± 1.35	0.386
hs-CRP (mg/L)	4.41 (2.71, 5.37)	4.28 (2.58, 5.32)	4.58 (2.83, 5.44)	0.098
SAA (mg/L)	5.40 (4.80, 6.70)	5.30 (4.80, 6.30)	5.50 (4.90, 7.10)	<0.001
Ferritin (ng/mL)	127.90 (37.15, 227.20)	200.20 (134.20, 304.20)	42.30 (12.30, 118.50)	<0.001
TC (mmol/L)	4.46 ± 0.92	4.45 ± 0.92	4.48 ± 0.92	0.484
LDL-C (mmol/L)	2.65 ± 0.79	2.65 ± 0.79	2.64 ± 0.79	0.775
HDL-C (mmol/L)	1.28 ± 0.34	1.18 ± 0.34	1.37 ± 0.31	<0.001
TG (mmol/L)	0.93 (0.69, 1.37)	1.05 (0.78, 1.55)	0.84 (0.64, 1.17)	<0.001
ApoA1 (g/L)	1.24 ± 0.23	1.19 ± 0.23	1.29 ± 0.22	<0.001
ApoB (g/L)	0.74 ± 0.17	0.75 ± 0.17	0.72 ± 0.17	0.001
Lp(a) (mg/L)	86.00 (43.85, 200.05)	89.80 (44.80, 209.15)	81.35 (43.00, 185.60)	0.321
sdLDL-C (mg/L)	35.28 ± 15.84	37.63 ± 15.67	32.85 ± 15.65	<0.001

**Notes.**

Abbreviations N number WC waist circumference BMI body mass index SBP systolic blood pressure DBP dilation blood pressure FBG fast blood glucose HR heart rate hs-CRP high-sensitivity C-reactive protein SAA serum amyloid A TC total cholesterol LDL-C low-density lipoprotein cholesterol HDL-C high-density lipoprotein cholesterol TG triglycerides ApoA1 apolipoprotein A1 ApoB apolipoprotein B Lp(a) lipoprotein a sdLDL-C small dense LDL-C

Continuous variables were shown as means ± standard deviation.

Categorical variables were shown as numbers (percentages).

### Correlation between SF and atherogenic lipid profiles

**Figure 1:**
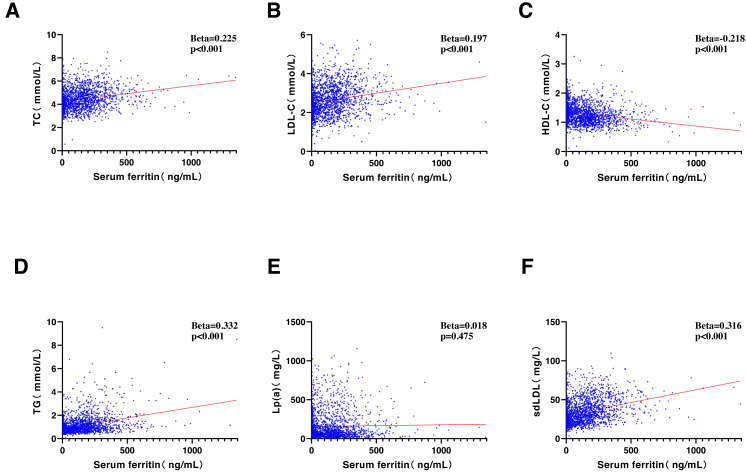
Correlative analyses were conducted between serum ferritin (ng/ml) and (A) TC (mmol/L), (B) LDL-C (mmol/L), (C) HDL-C (mmol/L), (D) TG (mmol/L), (E) Lp(a) (mg/L), and (F) sdLDL-C (mg/L). TC, total cholesterol; LDL-C, low-density lipoprotein cholesterol; HDL-C, high-density lipoprotein cholesterol; TG, triglycerides; Lp(a), lipoprotein a; sdLDL-C, small and dense LDL-C.

SF was significantly correlated with most of the lipids, including TC (Beta = 0.225, *P* < 0.001), LDL-C (Beta = 0.197, *P* < 0.001), HDL-C (Beta = −0.218, *P* < 0.001), TG (Beta = 0.332, *P* < 0.001), and sdLDL-C (Beta = 0.316, *P* < 0.001). An exception was observed with Lp(a), which showed no significant correlation (Beta = 0.018, *P* = 0.475) (Fig. 1).

### Sex-specific correlation between SF concentration and atherogenic lipids in different inflammatory states

SF was not significantly correlated with hs-CRP (*r* = 0.015, *P* = 0.537) and SAA (*r* = 0.004, *P* = 0.854), but there was a significant correlation between hs-CRP and SAA (*r* = 0.753, *P* < 0.001). To ascertain whether the correlation between SF and lipid profiles remained independent of the inflammatory states, subgroup analyses were conducted, stratifying the participants based on the interquartile range of hs-CRP and SAA levels. Given the significant difference in SF between men and women, sex-specific correlation analyses were performed. SF exhibited significant correlations with TC, TG, and sdLDL-C levels in all subgroups, in both men and women. In women, SF was significantly correlated with LDL-C and HDL-C in all subgroups. No significant correlation was observed between SF concentration and Lp(a) levels in any of the subgroups (Table 2).

### Association of SF concentration with different types of dyslipidemia

**Figure 2:**
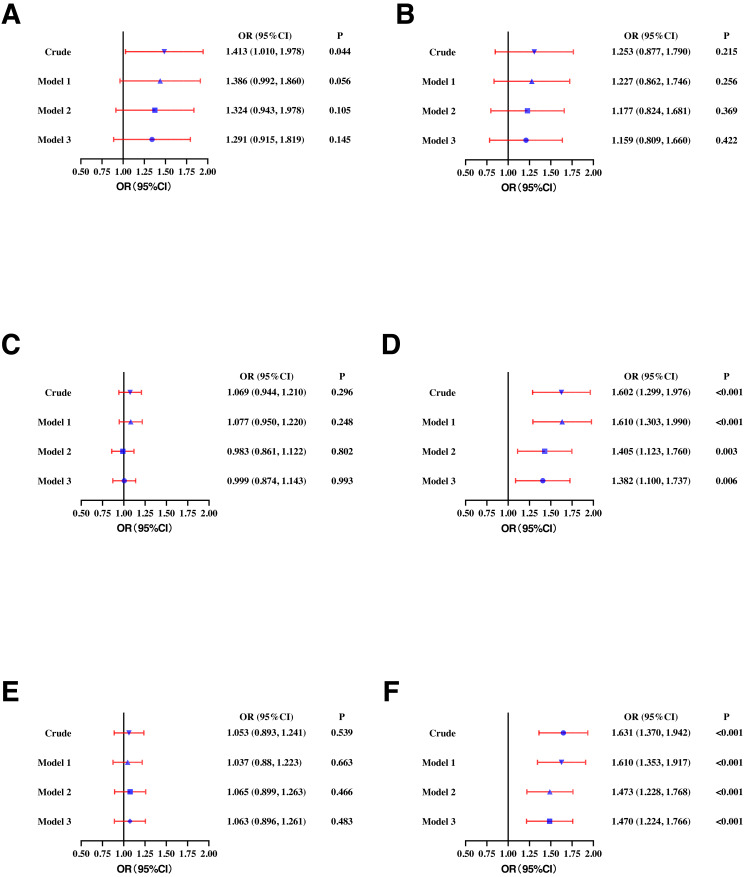
Odds ratios (ORs) and their 95% confidence intervals (95% CIs) for (A) high TC, (B) high LDL-C, (C) low HDL-C, (D) high TG, (E) high Lp(a), and (F) high sdLDL-C according to serum ferritin quartile in males. TC, total cholesterol; LDL-C, low-density lipoprotein cholesterol; HDL-C, high-density lipoprotein cholesterol; TG, triglycerides; Lp(a), lipoprotein a; sdLDL-C, small and dense LDL-C; Crude, ORs with no adjustments. Model 1: ORs adjusted for age. Model 2: ORs adjusted for waist circumference, BMI, and blood pressure based on model 1. Model 3: ORs adjusted for smoking, drinking and exercise based on model 2. Definitions of different types of dyslipidemia are described in the Methods section.

In the male population, higher SF was positively correlated with high TC (OR: 1.413, 95% CI [1.010–1.978]), high TG (OR: 1.602, 95% CI [1.299–1.976]), and high sdLDL-C (OR: 1.631, 95% CI [1.370–1.942]). After adjusting for age (model 1), SF maintained a significant correlation with high TG (OR: 1.610, 95% CI [1.303–1.990]) and high sdLDL-C (OR: 1.610, 95% CI [1.353–1.917]). In model 2 (adjusting for WC, BMI, and BP based on model 1) and model 3 (adjusting for smoking, drinking, and exercise based on model 2), SF concentration remained significantly correlated with high sdLDL-C (OR: 1.470, 95% CI [1.224–1.766]) and high TG (OR: 1.382, 95% CI [1.100–1.737]) (Fig. 2).

**Figure 3:**
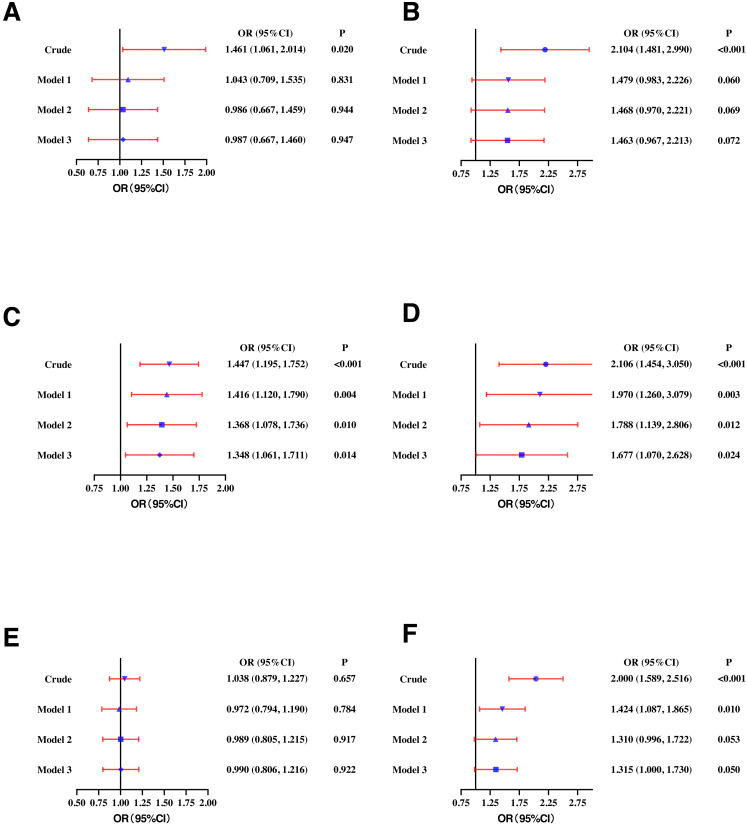
Odds ratios (ORs) and their 95% confidence intervals (95% CIs) for (A) high TC, (B) high LDL-C, (C) low HDL-C, (D) high TG, (E) high Lp(a), and (F) high sdLDL-C according to serum ferritin quartile in females. TC, total cholesterol. lipoprotein cholesterol; HDL-C, high-density lipoprotein cholesterol; TG, triglycerides; Lp(a), lipoprotein a; sdLDL-C, small and dense LDL-C; Crude, ORs with no adjustments. Model 1: ORs adjusted for age. Model 2: ORs adjusted for waist circumference, BMI, and blood pressure based on model 1. Model 3: ORs adjusted for smoking, drinking and exercise based on model 2. Definitions of different types of dyslipidemia are described in the Methods section.

In the female population, higher SF was positively correlated with high TC (OR: 1.461, 95% CI [1.061–2.014]), high LDL-C (OR: 2.104, 95% CI [1.481–2.990]), low HDL-C (OR: 1.447, 95% CI [1.195–1.752]), high TG (OR: 2.106, 95% CI [1.454–3.050]), and high sdLDL-C (OR: 2.000, 95% CI [1.589–2.516]). In model 1, higher SF was significantly correlated with high TG (OR: 1.970, 95% CI [1.260–3.079]), low HDL-C (OR: 1.416, 95% CI [1.120–1.790]), and high sdLDL-C (OR: 1.424, 95% CI [1.087–1.865]). In model 2 and model 3, higher SF concentration remained significantly correlated with low HDL-C (OR: 1.348, 95% CI [1.061–1.711]) and high TG (OR: 1.677, 95% CI [1.070–2.628]) (Fig. 3).

**Table 2:** Sex specific correlations between serum ferritin concentrations and lipid profiles stratified by hs-CRP and SAA levels.

	TC (mmol/L)		LDL-C (mmol/L)		HDL-C (mmol/L)		TG (mmol/L)		Lp(a) (mg/L)		sdLDL-C (mg/L)	
	Beta	*P*	Beta	*P*	Beta	*P*	Beta	*P*	Beta	*P*	Beta	*P*
Men												
hs-CRP (mg/L)												
Q1	0.245	<0.001	0.066	0.333	−0.055	0.427	0.334	<0.001	0.007	0.922	0.154	0.024
Q2	0.290	<0.001	0.231	0.001	0.004	0.955	0.168	0.013	−0.057	0.405	0.309	<0.001
Q3	0.139	0.044	0.052	0.454	0.060	0.383	0.185	0.007	−0.061	0.381	0.177	0.010
Q4	0.186	0.005	0.155	0.021	−0.069	0.306	0.222	0.001	0.134	0.050	0.231	0.001
SAA (mg/L)												
Q1	0.295	<0.001	0.250	<0.001	−0.003	0.965	0.212	0.003	0.025	0.725	0.289	<0.001
Q2	0.133	0.045	0.089	0.180	−0.087	0.192	0.202	0.002	0.004	0.954	0.201	0.002
Q3	0.194	0.005	−0.011	0.875	−0.110	0.110	0.375	<0.001	−0.110	0.114	0.151	0.028
Q4	0.249	<0.001	0.176	0.008	0.078	0.243	0.164	0.013	0.062	0.361	0.241	<0.001
Women												
hs-CRP (mg/L)												
Q1	0.324	<0.001	0.349	<0.001	−0.254	<0.001	0.361	<0.001	−0.072	0.307	0.369	<0.001
Q2	0.307	<0.001	0.327	<0.001	−0.193	0.005	0.275	<0.001	0.091	0.193	0.297	<0.001
Q3	0.373	<0.001	0.409	<0.001	−0.216	0.002	0.275	<0.001	0.024	0.729	0.414	<0.001
Q4	0.279	<0.001	0.313	<0.001	−0.219	0.001	0.337	<0.001	−0.043	0.546	0.341	<0.001
SAA (mg/L)												
Q1	0.359	<0.001	0.355	<0.001	−0.247	0.001	0.381	<0.001	−0.072	0.362	0.453	<0.001
Q2	0.375	<0.001	0.390	<0.001	−0.288	<0.001	0.376	<0.001	0.115	0.087	0.410	<0.001
Q3	0.340	<0.001	0.369	<0.001	−0.201	0.002	0.299	<0.001	−0.006	0.930	0.329	<0.001
Q4	0.188	0.006	0.263	<0.001	−0.275	<0.001	0.248	<0.001	−0.035	0.618	0.271	<0.001

**Notes.**

hs-CRP high-sensitivity C-reactive protein SAA serum amyloid A TC total cholesterol LDL-C low-density lipoprotein cholesterol HDL-C high-density lipoprotein cholesterol TG triglycerides Lp(a) lipoprotein a sdLDL-C small dense LDL-C Q1 first quartile Q2 second quartile Q3 third quartile Q4 fourth quartile Beta standardized regression coefficients *P* *p* values

### Subgroup analysis for young (18–35 years) and middle-aged (≥45 years) participants

In the young participants group, SF was significantly correlated with TC (Beta = 0.192, *P* < 0.001), LDL-C (Beta = 0.168, *P* < 0.001), HDL-C (Beta = −0.313, *P* < 0.001), TG (Beta = 0.374, *P* < 0.001), and sdLDL-C (Beta = 0.366, *P* < 0.001). There was no correlation between SF and Lp(a) (Beta = −0.008, *P* = 0.845) (Fig. S1). In the middle-aged participants group, SF was significantly correlated with TC (Beta = 0.162, *P* < 0.001), LDL-C (Beta = 0.110, *P* = 0.005), HDL-C (Beta = −0.162, *P* < 0.001), TG (Beta = 0.299, *P* < 0.001), and sdLDL-C (Beta = 0.214, *P* < 0.001). There was no correlation between SF and Lp(a) (Beta = −0.001, *P* = 0.977) (Fig. S2).

**Figure 4:**
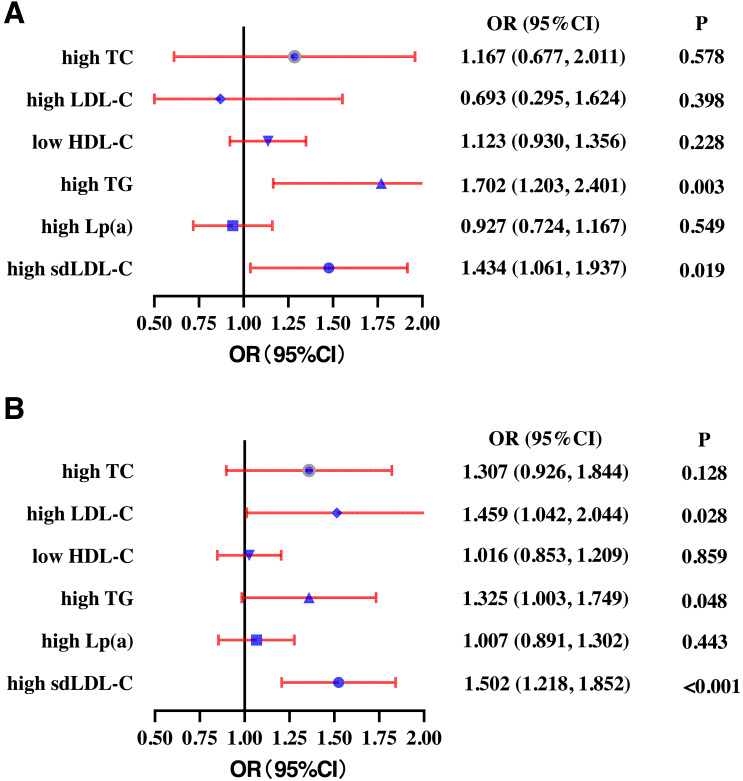
Subgroup analysis of the odds ratios (ORs) of different types of dyslipidemia according to serum ferritin quartiles for young (18-35 years) (A) and middle-aged (≥ 45 years) (B) participants. High TC, high total cholesterol; High LDL-C, high low-density lipoprotein cholesterol; Low HDL-C, low high-density lipoprotein cholesterol. High TG, high triglycerides; High Lp(a), high lipoprotein a. High sdLDL-C, small and dense LDL-C. Definitions of different types of dyslipidemia are described in the Methods section.

In the young population, higher SF quartile was positively correlated with high TG (OR: 1.702, 95% CI [1.203–2.407]) and high sdLDL-C (OR: 1.434, 95% CI [1.061–1.937]). There was no correlation between SF quartile and high TC (OR: 1.167, 95% CI [0.677–2.011]), high LDL-C (OR: 0.693, 95% CI [0.295–1.624]), low HDL-C (OR: 1.123 95% CI [0.930–1.356]), and high Lp(a) (OR: 0.927, 95% CI [0.724–1.167]) after adjusting for gender, WC, BMI, SBP, smoking, drinking, and exercise (Fig. 4A). In the middle-aged participants group, higher SF quartile was positively correlated with high LDL-C (OR: 1.459, 95% CI [1.042–2.044]), high TG (OR: 1.325, 95% CI [1.003–1.749]), and high sdLDL-C (OR: 1.502, 95% CI [1.218–1.852]). There was no correlation between SF quartile and high TC (OR: 1.307, 95% CI [0.926–1.844]), low HDL-C (OR: 1.016 95% CI [0.853–1.209]), or high Lp(a) (OR: 1.007, 95% CI [0.891–1.302]) (Fig. 4B).

## Discussion

In this study, it was found that SF concentration was significantly higher in men compared to women. SF exhibited positively correlations with TC, LDL-C, TG, and sdLDL-C. Conversely, it displayed a negatively correlation with HDL-C. There was no significant correlation with Lp(a). Under different inflammatory conditions, SF was positively correlated with LDL-C and negatively correlated with HDL-C in women. SF was positively correlated with TC, TG, and sdLDL-C in both men and women. Higher SF levels were associated with high TC, high TG, and high sdLDL-C in men, and almost all kinds of dyslipidemia except for high Lp(a) in women. Even after adjusting for confounders, higher SF levels remained significantly associated with high TG in both men and women. The correlations between SF and TC, LDL-C, HDL-C, TG, and sdLDL-C were still significant in both young and middle-aged subgroups.

People who have long-term residence at high altitudes are in a hypoxic environment. Hypoxemia is sensed by prolyl hydroxylases (PHDs), which stabilize hypoxia-inducible factor (HIF). HIF can promote the expression of erythropoietin (Epo), increasing red blood cell production (Gassmann & Muckenthaler, 2015). Epo, hepcidin, and erythroferrone are involved in coordinating the liver, bone marrow, and small intestine, as well as altering iron acquisition. Therefore, altitude is closely related to iron metabolism. In this study, the mean Hb levels of high-altitude Tajik were up to 168.17 g/L in males and 149.28 g/l in females, indicating a high-altitude adaptation. Several studies conducted in different populations living at high altitudes showed that iron stores were distinct in healthy individuals and vulnerable populations with increased iron demand (Böning et al., 2004; Burke et al., 2017; Cook et al., 2005; Tufts et al., 1985). We reported that the median SF of Tajik males and females were 200.20 ng/mL and 42.30 ng/mL, respectively. Compared to the study conducted on Bolivian adults living at 3,500 m of altitude or above, the SF levels of male Tajik were much higher, but the SF levels of female Tajiks were similar (Beall et al., 1990). Regrettably, we have no data on SF in low-altitude Tajik populations since most Tajiks live on the high-altitude Pamirs Plateau of China, which prevented us from investigating the effects of high altitude on iron store.

Previous research indicated a consistent pattern regarding the relationship between SF and lipid profiles, showing positive correlations with TC, LDL-C, and TG, while revealing negative correlation with HDL-C levels in populations from the United States (Li et al., 2023) and Middle East (Al Akl et al., 2021). In this study conducted among high-altitude Tajik individuals, these trends were similarly observed. Notably, serum Lp(a) levels are mainly determined by genetic factors (Kronenberg et al., 2022; Reyes-Soffer et al., 2022), and the prevalence of high Lp(a) remained unassociated with SF in both men and women in this study. The exact way in which SF concentrations are related to dyslipidemia has not been fully grasped yet. Numerous studies have proven that SF was associated with other CVD risk factors, such as diabetes (Sun et al., 2013), hypertension (Piperno et al., 2002), elevated fasting insulin and blood glucose (Tuomainen et al., 1997), central adiposity (Gillum, 2001), and metabolic syndrome (Jehn, Clark & Guallar, 2004; Suárez-Ortegón et al., 2018), suggesting that SF may play a significant role in metabolism. SF could lose its iron and the unliganded iron has the capacity to catalyze hydroxyl radical formation, leading to oxidative tissue damage (Kell & Pretorius, 2014; Wolff, 1993). Studies on rats showed that dietary iron restriction can lead to decreased TG concentrations and reduced levels of lipid peroxidation levels (Vargas-Vargas et al., 2022). However, iron overload may induce elevated TG levels through several mechanisms, including enhanced hepatic oxidative stress, decreased fatty acid beta-oxidation, and the promoted hepatic lipid secretion by increasing the expression of apoB-100 and microsomal triglyceride transfer protein (Silva et al., 2015).

SF, apart from being a sign of iron storage, serves as a crucial marker for inflammatory diseases too, since it is mainly a product leaked from damaged cells (Kell & Pretorius, 2014). Therefore, is the relationship between SF and lipid profiles in high-altitude residents associated with its role as an inflammation marker? A previous study conducted in the general Chinese population found that the association between SF and lipid parameters persisted after adjusting for CRP levels, suggesting that low-grade inflammation may not be the sole explanation for the link between SF levels and dyslipidemia (Li et al., 2017). In this study, individuals with chronic inflammatory diseases were excluded. Notably, SF showed no significant correlation with two other inflammation markers, hsCRP and SAA. Moreover, SF remained significantly associated with TC, TG, and sdLDL-C across subgroups stratified by varying levels of hs-CRP and SAA. These findings suggest that the relationship between SF and lipid levels cannot be attributed to its role as an inflammation marker.

There seemed to be a gender discrepancy in the association between SF and lipids levels or dyslipidemia. A study conducted on Korean adolescents found that SF was positively associated with TC, LDL-C, and TG levels, and negatively associated with HDL-C levels in women. However, in men, the association was observed only with HDL-C levels (Kim et al., 2016). This result differed from a study conducted in US adults, where only females showed a clearly increasing tendency for high-TC and high-LDL-C. In this study, the relationship between SF and lipid levels appeared to be stronger in women, which aligned with the findings from the US study. In women, five out of six lipid parameters were correlated with SF across varying levels of inflammation. In contrast, only three out of six lipid parameters were significantly associated with SF in men. Regarding dyslipidemia, nearly all types of dyslipidemia, except for high Lp(a), were associated with higher SF in women. However, after adjusting for confounders, SF was only associated with high TG in both men and women, indicating that the gender discrepancy in the association between SF and lipids might be attributed to differences in the distributions of confounders between men and women.

There are a number of limitations in this study which need to be taken into account. First, because of its cross-sectional nature, the study was unable to determine a causal connection between SF and lipid profiles. Prospective cohort studies would be necessary to determine a causal relationship. Second, although this study was conducted in a single ethnic group with similar eating habits, dietary information was not included in the study, making it challenging to adjust for dietary factors (especially iron intake) in the analysis of the relationship between SF and lipids. Third, the menstrual status of women was unknown, which might have introduced potential bias to the results.

Despite these limitations, a key strength of this study lies in its novel investigation of the association between SF and lipid profiles under varying inflammatory conditions in high-altitude living residents. Moreover, as far as we know, this is the first study to look into the relation between SF and Lp(a) as well as sdLDL-C. These findings help us better understand the interplay between SF and lipids, providing useful perspectives and create a basis for more researches in the future.

In conclusion, SF was positively correlated with TC, LDL-C, TG, and sdLDL-C; negatively correlated with HDL-C; and not significantly correlated with Lp(a). Even after adjusting for confounders, higher SF levels were associated with high TG in both genders. The relationship between SF and lipid levels cannot be attributed to its role as an inflammation marker.

##  Supplemental Information

10.7717/peerj.19104/supp-1 Supplemental Information 1 STROBE checklist

10.7717/peerj.19104/supp-2 Supplemental Information 2 Correlative analyses were conducted between serum ferritin (ng/ml) and A: TC (mmol/L), B: LDL-C (mmol/L), C: HDL-C (mmol/L), D: TG (mmol/L), E: Lp(a) (mg/L), and F: sdLDL-C (mg/L) in young (18-35 years) participants TC: total cholesterol. LDL-C: low-density lipoprotein cholesterol. HDL-C: high-density lipoprotein cholesterol. TG: triglycerides. Lp(a): lipoprotein a. sdLDL-C: small and dense LDL-C.

10.7717/peerj.19104/supp-3 Supplemental Information 3 Correlative analyses were conducted between serum ferritin (ng/ml) and A: TC (mmol/L), B: LDL-C (mmol/L), C: HDL-C (mmol/L), D: TG (mmol/L), E: Lp(a) (mg/L), and F: sdLDL-C (mg/L) in middle-aged (≥ 45 years) participants TC: total cholesterol. LDL-C: low-density lipoprotein cholesterol. HDL-C: high-density lipoprotein cholesterol. TG: triglycerides. Lp(a): lipoprotein a. sdLDL-C: small and dense LDL-C.

10.7717/peerj.19104/supp-4 Supplemental Information 4 Raw data

## Additional Information and Declarations

### Data Availability

The following information was supplied regarding data availability: The raw measurements are available in the Supplementary Files.

## References

[ref-1] Al Akl NS, Khalifa O, Errafii K, Arredouani A (2021). Association of dyslipidemia, diabetes and metabolic syndrome with serum ferritin levels: a middle eastern population-based cross-sectional study. Scientific Reports.

[ref-2] Alberti KG, Zimmet PZ (1998). Definition, diagnosis and classification of diabetes mellitus and its complications. Part 1: diagnosis and classification of diabetes mellitus provisional report of a WHO consultation. Diabetic Medicine.

[ref-3] Arnett DK, Blumenthal RS, Albert MA, Buroker AB, Goldberger ZD, Hahn EJ, Himmelfarb CD, Khera A, Lloyd-Jones D, McEvoy JW, Michos ED, Miedema MD, Muñoz D, SmithJr SC, Virani SS, WilliamsSr KA, Yeboah J, Ziaeian B (2019). 2019 ACC/AHA guideline on the primary prevention of cardiovascular disease: a report of the american college of cardiology/american heart association task force on clinical practice guidelines. Circulation.

[ref-4] Beall CM, Brittenham GM, Macuaga F, Barragan M (1990). Variation in hemoglobin concentration among samples of high-altitude natives in the Andes and the Himalayas. American Journal of Human Biology.

[ref-5] Böning D, Cristancho E, Serrato M, Reyes O, Mora M, Coy L, Rojas J (2004). Hemoglobin mass and peak oxygen uptake in untrained and trained female altitude residents. International Journal of Sports Medicine.

[ref-6] Burke RM, Rebolledo PA, Fabiszewski De Aceituno AM, Revollo R, Iñiguez V, Klein M, Drews-Botsch C, Leon JS, Suchdev PS (2017). Early deterioration of iron status among a cohort of Bolivian infants. Maternal & Child Nutrition.

[ref-7] Cook JD, Boy E, Flowers C, Daroca M del C (2005). The influence of high-altitude living on body iron. Blood.

[ref-8] Dhindsa DS, Sandesara PB, Shapiro MD, Wong ND (2020). The evolving understanding and approach to residual cardiovascular risk management. Frontiers in Cardiovascular Medicine.

[ref-9] Gassmann M, Muckenthaler MU (2015). Adaptation of iron requirement to hypoxic conditions at high altitude. Journal of Applied Physiology.

[ref-10] Gillum RF (2001). Association of serum ferritin and indices of body fat distribution and obesity in Mexican American men—the Third National Health and Nutrition Examination Survey. International Journal of Obesity and Related Metabolic Disorders.

[ref-11] González AS, Guerrero DB, Soto MB, Díaz SP, Martinez-Olmos M, Vidal O (2006). Metabolic syndrome, insulin resistance and the inflammation markers C-reactive protein and ferritin. European Journal of Clinical Nutrition.

[ref-12] Jehn M, Clark JM, Guallar E (2004). Serum ferritin and risk of the metabolic syndrome in U.S. adults. Diabetes Care.

[ref-13] Kell DB, Pretorius E (2014). Serum ferritin is an important inflammatory disease marker, as it is mainly a leakage product from damaged cells. Metallomics.

[ref-14] Kim YE, Kim DH, Roh YK, Ju SY, Yoon YJ, Nam GE, Nam HY, Choi JS, Lee JE, Sang JE, Han K, Park YG (2016). Relationship between Serum Ferritin Levels and Dyslipidemia in Korean Adolescents. PLOS ONE.

[ref-15] Krauss RM (2022). Small dense low-density lipoprotein particles: clinically relevant?. Current Opinion in Lipidology.

[ref-16] Kronenberg F, Mora S, Stroes ESG, Ference BA, Arsenault BJ, Berglund L, Dweck MR, Koschinsky M, Lambert G, Mach F, McNeal CJ, Moriarty PM, Natarajan P, Nordestgaard BG, Parhofer KG, Virani SS, Eckardstein Avon, Watts GF, Stock JK, Ray KK, Tokgözoğlu LS, Catapano AL (2022). Lipoprotein(a) in atherosclerotic cardiovascular disease and aortic stenosis: a European Atherosclerosis Society consensus statement. European Heart Journal.

[ref-17] Lawler PR, Bhatt DL, Godoy LC, Lüscher TF, Bonow RO, Verma S, Ridker PM (2021). Targeting cardiovascular inflammation: next steps in clinical translation. European Heart Journal.

[ref-18] Li G, Yu W, Yang H, Wang X, Ma T, Luo X (2023). Relationship between Serum Ferritin Level and Dyslipidemia in US adults based on data from the national health and nutrition examination surveys 2017 to 2020. Nutrients.

[ref-19] Li J, Bao W, Zhang T, Zhou Y, Yang H, Jia H, Wang R, Cao Y, Xiao C (2017). Independent relationship between serum ferritin levels and dyslipidemia in Chinese adults: a population study. PLOS ONE.

[ref-20] Joint Committee on the Chinese Guidelines for Lipid Management (2023). Guidelines for lipid management, Chinese guidelines for lipid management (2023). Chinese Journal of Cardiology.

[ref-21] Liu YS, Huang H, Zhou SM, Tian HJ, Li P (2018). Excessive iron availability caused by disorders of Interleukin-10 and Interleukin-22 contributes to high altitude polycythemia. Frontiers in Physiology.

[ref-22] Lopez A, Cacoub P, Macdougall IC, Peyrin-Biroulet L (2016). Iron deficiency anaemia. Lancet.

[ref-23] Mach F, Baigent C, Catapano AL, Koskinas KC, Casula M, Badimon L, Chapman MJ, De Backer GG, Delgado V, Ference BA, Graham IM, Halliday A, Landmesser U, Mihaylova B, Pedersen TR, Riccardi G, Richter DJ, Sabatine MS, Taskinen M-R, Tokgozoglu L, Wiklund O, Mueller C, Drexel H, Aboyans V, Corsini A, Doehner W, Farnier M, Gigante B, Kayikcioglu M, Krstacic G, Lambrinou E, Lewis BS, Masip J, Moulin P, Petersen S, Petronio AS, Piepoli MF, Pintó X, Räber L, Ray KK, Reiner Ž, Riesen WF, Roffi M, Schmid J-P, Shlyakhto E, Simpson IA, Stroes E, Sudano I, Tselepis AD, Viigimaa M, Vindis C, Vonbank A, Vrablik M, Vrsalovic M, Zamorano JL, Collet J-P, Koskinas KC, Casula M, Badimon L, John Chapman M, De Backer GG, Delgado V, Ference BA, Graham IM, Halliday A, Landmesser U, Mihaylova B, Pedersen TR, Riccardi G, Richter DJ, Sabatine MS, Taskinen M-R, Tokgozoglu L, Wiklund O, Windecker S, Aboyans V, Baigent C, Collet J-P, Dean V, Delgado V, Fitzsimons D, Gale CP, Grobbee D, Halvorsen S, Hindricks G, Iung B, Jüni P, Katus HA, Landmesser U, Leclercq C, Lettino M, Lewis BS, Merkely B, Mueller C, Petersen S, Petronio AS, Richter DJ, Roffi M, Shlyakhto E, Simpson IA, Sousa-Uva M, Touyz RM, Nibouche D, Zelveian PH, Siostrzonek P, Najafov R, Van de Borne P, Pojskic B, Postadzhiyan A, Kypris L, Špinar J, Larsen ML, Eldin HS, Viigimaa M, Strandberg TE, Ferrières J, Agladze R, Laufs U, Rallidis L, Bajnok L, Gudjónsson T, Maher V, Henkin Y, Gulizia MM, Mussagaliyeva A, Bajraktari G, Kerimkulova A, Latkovskis G, Hamoui O, Slapikas R, Visser L, Dingli P, Ivanov V, Boskovic A, Nazzi M, Visseren F, Mitevska I, Retterstøl K, Jankowski P, Fontes-Carvalho R, Gaita D, Ezhov M, Foscoli M, Giga V, Pella D, Fras Z, De Isla LP, Hagström E, Lehmann R, Abid L, Ozdogan O, Mitchenko O, Patel RS (2020). 2019 ESC/EAS Guidelines for the management of dyslipidaemias: lipid modification to reduce cardiovascular risk. European Heart Journal.

[ref-24] Piperno A, Trombini P, Gelosa M, Mauri V, Pecci V, Vergani A, Salvioni A, Mariani R, Mancia G (2002). Increased serum ferritin is common in men with essential hypertension. Journal of Hypertension.

[ref-25] Reyes-Soffer G, Ginsberg HN, Berglund L, Duell PB, Heffron SP, Kamstrup PR, Lloyd-Jones DM, Marcovina SM, Yeang C, Koschinsky ML (2022). Lipoprotein(a): a genetically determined, causal, and prevalent risk factor for atherosclerotic cardiovascular disease: a scientific statement from the american heart association. Arteriosclerosis, Thrombosis, and Vascular Biology.

[ref-26] Silva M, da Costa Guerra JF, Sampaio AF, De Lima WG, Silva ME, Pedrosa ML (2015). Iron dextran increases hepatic oxidative stress and alters expression of genes related to lipid metabolism contributing to hyperlipidaemia in murine model. BioMed Research International.

[ref-27] Staub K, Haeusler M, Bender N, Morozova I, Eppenberger P, Panczak R, Zwahlen M, Schaer DJ, Maggiorini M, Ulrich S, Gassmann NN, Muckenthaler MU, Rühli F, Gassmann M (2020). Hemoglobin concentration of young men at residential altitudes between 200 and 2000 m mirrors Switzerland’s topography. Blood.

[ref-28] Suárez-Ortegón MF, Ensaldo-Carrasco E, Shi T, McLachlan S, Fernández-Real JM, Wild SH (2018). Ferritin, metabolic syndrome and its components: a systematic review and meta-analysis. Atherosclerosis.

[ref-29] Sun L, Zong G, Pan A, Ye X, Li H, Yu Z, Zhao Y, Zou S, Yu D, Jin Q, Hu FB, Lin X (2013). Elevated plasma ferritin is associated with increased incidence of type 2 diabetes in middle-aged and elderly Chinese adults. Journal of Nutrition.

[ref-30] Tufts DA, Haas JD, Beard JL, Spielvogel H (1985). Distribution of hemoglobin and functional consequences of anemia in adult males at high altitude. American Journal of Clinical Nutrition.

[ref-31] Tuomainen TP, Nyyssönen K, Salonen R, Tervahauta A, Korpela H, Lakka T, Kaplan GA, Salonen JT (1997). Body iron stores are associated with serum insulin and blood glucose concentrations, population study in 1013 eastern finnish men. Diabetes Care.

[ref-32] Vargas-Vargas MA, Saavedra-Molina A, Gómez-Barroso M, Peña Montes D, Cortés-Rojo C, Miguel H, Trujillo X, Montoya-Pérez R (2022). Dietary iron restriction improves muscle function, dyslipidemia, and decreased muscle oxidative stress in streptozotocin-induced diabetic rats. Antioxidants.

[ref-33] Visseren FLJ, Mach F, Smulders YM, Carballo D, Koskinas KC, Bäck M, Benetos A, Biffi A, Boavida JM, Capodanno D, Cosyns B, Crawford C, Davos CH, Desormais I, Di Angelantonio E, Franco OH, Halvorsen S, Hobbs FDR, Hollander M, Jankowska EA, Michal M, Sacco S, Sattar N, Tokgozoglu L, Tonstad S, Tsioufis KP, Van Dis I, Van Gelder IC, Wanner C, Williams B (2021). 2021 ESC Guidelines on cardiovascular disease prevention in clinical practice. European Heart Journal.

[ref-34] Wolff SP (1993). Diabetes mellitus and free radicals, Free radicals, transition metals and oxidative stress in the aetiology of diabetes mellitus and complications. British Medical Bulletin.

[ref-35] Yanai H, Adachi H, Hakoshima M, Katsuyama H (2022). Atherogenic lipoproteins for the statin residual cardiovascular disease risk. International Journal of Molecular Sciences.

[ref-36] Zhang Y, Zhang J, Sheng H, Li H, Wang R (2019). Acute phase reactant serum amyloid A in inflammation and other diseases. Advances in Clinical Chemistry.

[ref-37] Zhou B, Liu S, Yuan G (2021). Combined associations of serum ferritin and body size phenotypes with cardiovascular risk profiles: a Chinese population-based study. Frontiers in Public Health.

[ref-38] Zhu Y, He B, Xiao Y, Chen Y (2019). Iron metabolism and its association with dyslipidemia risk in children and adolescents: a cross-sectional study. Lipids in Health and Disease.

