# Supplementary figures and images for: Serum ferritin associated with atherogenic lipid profiles in a high-altitude living general population
Source: PeerJ. 2025 Mar 24;13:e19104. doi: 10.7717/peerj.19104 (PMC11949108; doi:10.7717/peerj.19104)

**A**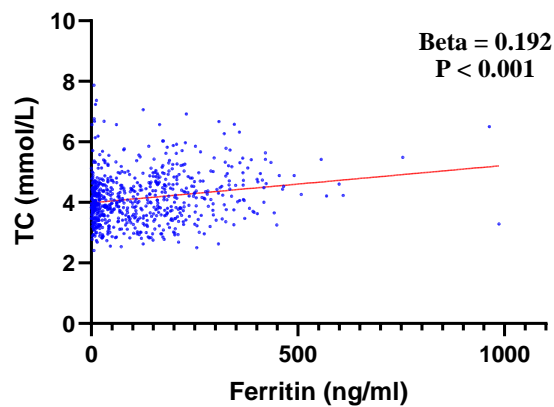**B**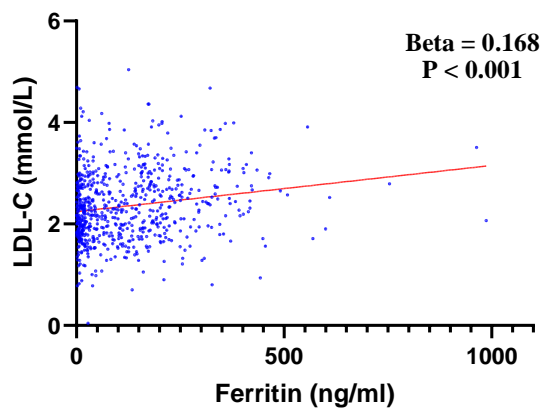**C**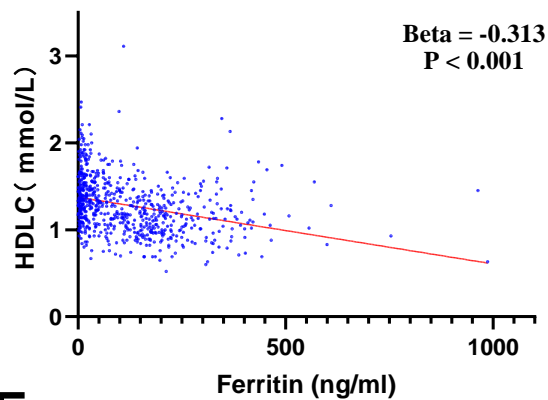**D**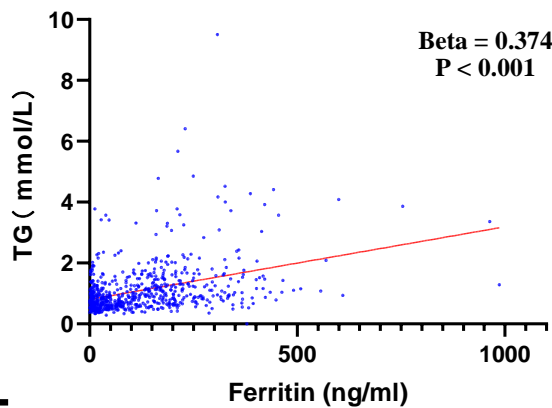**E**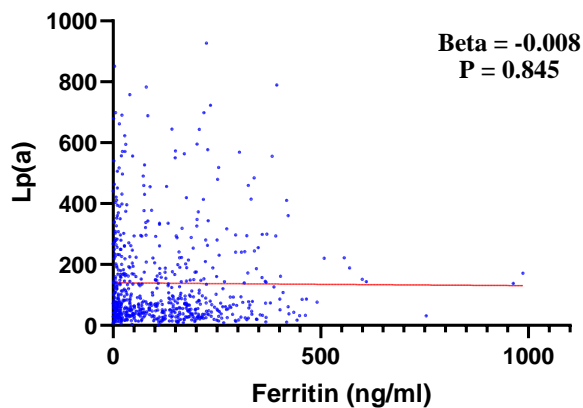**F**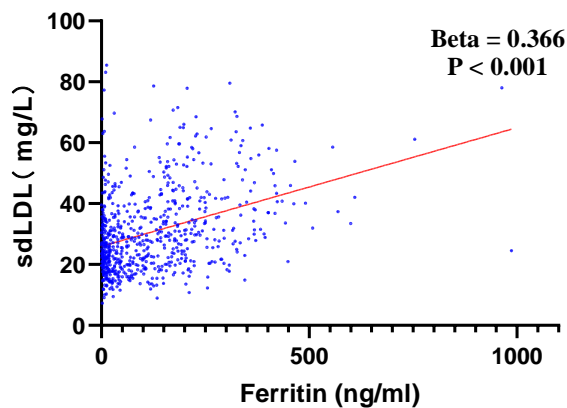

Supplement: Supplemental Information 2 — TC: total cholesterol. LDL-C: low-density lipoprotein cholesterol. HDL-C: high-density lipoprotein cholesterol. TG: triglycerides. Lp(a): lipoprotein a. sdLDL-C: small and dense LDL-C. [file peerj-13-19104-s002.pdf]

**A**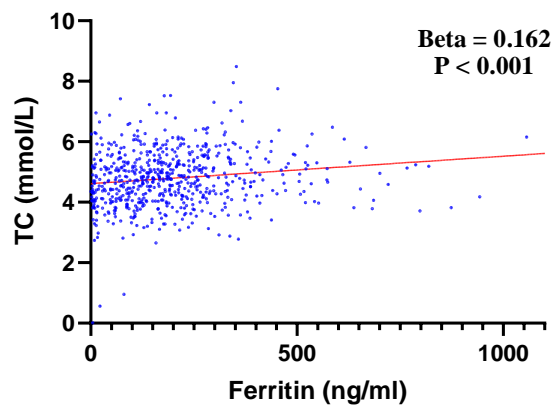**B**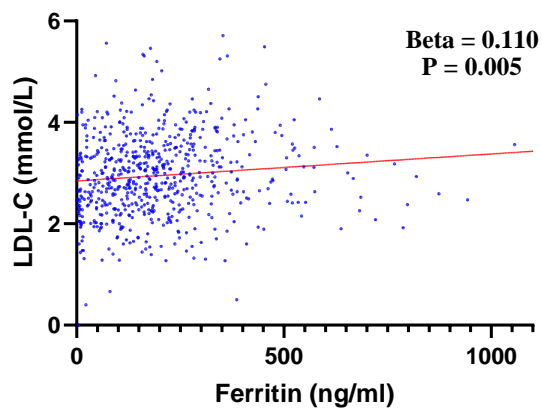**C**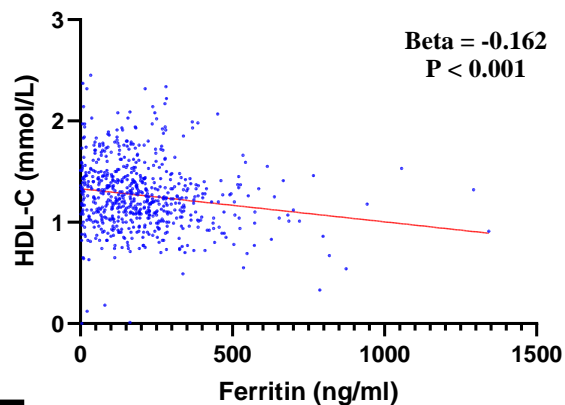**D**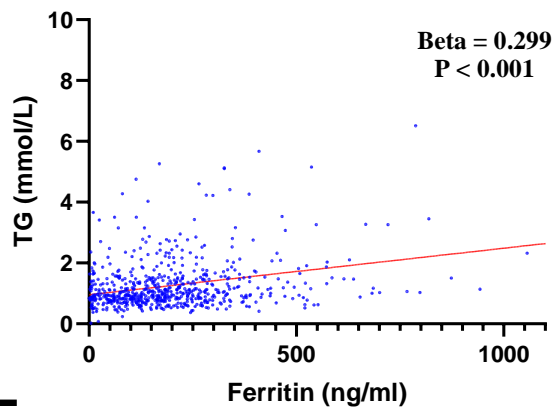**E**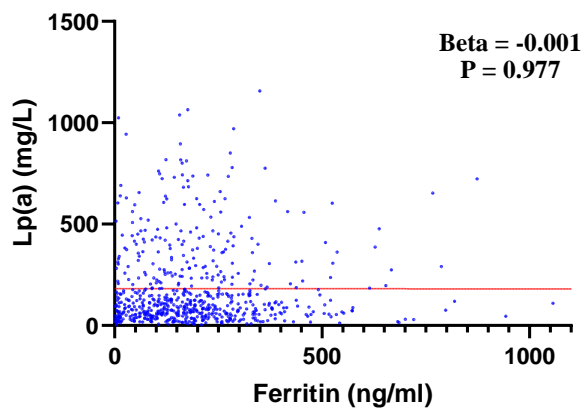**F**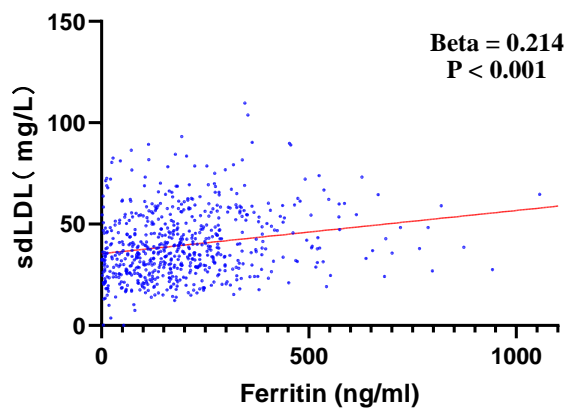

Supplement: Supplemental Information 3 — TC: total cholesterol. LDL-C: low-density lipoprotein cholesterol. HDL-C: high-density lipoprotein cholesterol. TG: triglycerides. Lp(a): lipoprotein a. sdLDL-C: small and dense LDL-C. [file peerj-13-19104-s003.pdf]
